# Hybrid Time-Dependent Ginzburg–Landau Simulations of Block Copolymer Nanocomposites: Nanoparticle Anisotropy

**DOI:** 10.3390/polym14091910

**Published:** 2022-05-07

**Authors:** Javier Diaz, Marco Pinna, Andrei V. Zvelindovsky, Ignacio Pagonabarraga

**Affiliations:** 1 CECAM, Centre Européen de Calcul Atomique et Moléculaire, EPFL, École Polytechnique Fédérale de Lausanne, Batochime—Avenue Forel 2, 1015 Lausanne, Switzerland; javier.diazbranas@epfl.ch (J.D.); ignacio.pagonabarraga@epfl.ch (I.P.); 2 Centre for Computational Physics, University of Lincoln, Brayford Pool, Lincoln LN6 7TS, UK; mpinna@lincoln.ac.uk; 3 Departament de Física de la Matèria Condensada, Universitat de Barcelona, Martí i Franqués 1, 08028 Barcelona, Spain; 4 Universitat de Barcelona Institute of Complex Systems (UBICS), Universitat de Barcelona, 08028 Barcelona, Spain

**Keywords:** block copolymer, nanoparticle, nanorod, colloid, nanocomposite, computer simulation, polymer, soft matter, hybrid material

## Abstract

Block copolymer melts are perfect candidates to template the position of colloidal nanoparticles in the nanoscale, on top of their well-known suitability for lithography applications. This is due to their ability to self-assemble into periodic ordered structures, in which nanoparticles can segregate depending on the polymer–particle interactions, size and shape. The resulting coassembled structure can be highly ordered as a combination of both the polymeric and colloidal properties. The time-dependent Ginzburg–Landau model for the block copolymer was combined with Brownian dynamics for nanoparticles, resulting in an efficient mesoscopic model to study the complex behaviour of block copolymer nanocomposites. This review covers recent developments of the time-dependent Ginzburg–Landau/Brownian dynamics scheme. This includes efforts to parallelise the numerical scheme and applications of the model. The validity of the model is studied by comparing simulation and experimental results for isotropic nanoparticles. Extensions to simulate nonspherical and inhomogeneous nanoparticles are discussed and simulation results are discussed. The time-dependent Ginzburg–Landau/Brownian dynamics scheme is shown to be a flexible method which can account for the relatively large system sizes required to study block copolymer nanocomposite systems, while being easily extensible to simulate nonspherical nanoparticles.

## 1. Introduction

Polymer nanocomposite materials composed of a polymer matrix and nanoparticle (NP) additives have attracted considerable attention due to applications where the polymer properties can be enhanced in the presence of nanoscopic fillers [1,2,3,4,5,6,7,8,9,10,11,12] (e.g., carbon black [13], electric field threshold reduction [14], flame-retardant applications [15] and photonic-gap materials [16,17,18,19]). Conversely, polymers and polymer blends remain an excellent choice to disperse NPs and control their interactions and locations [20]. Block copolymer (BCP) melts are fascinating materials due to their ability to self-assemble into ordered periodic structures in the nanoscale [21]. BCP melts can self-assemble into morphologies including body-centered cubic (BCC) spheres, hexagonally (HEX) ordered cylinders, gyroid and alternating lamellar. Each of these phases has a defined symmetry, as a consequence of the heterogeneity of the BCP chain, where monomers of chemically different kinds are grouped into blocks. This makes BCP melts ideal for applications where a precise spatial control in the nanoscale is required, such as the production of masks for nanolithography [22,23,24,25]. The phase behaviour of BCP melts in bulk and thin films have been widely studied [21,23,26,27,28,29,30,31]. In this work, we review recent hybrid time-dependent Ginzurg–Landau (TDGL) models coupled with Brownian dynamics (BD) for simulations of BCP nancomposites and their applications.

The well-ordered structures in which BCP melts can self-assemble serve as a perfect template to control the position of colloidal NPs given the intrinsic properties of symmetry of the BCP matrix [6,32,33]. Control over the placement of NPs within the BCP matrix was achieved by modifying the coating of the NP surface by grafting polymer chains on the surface [34,35,36,37,38,39]. The chemical interaction between the grafted chains and the BCP medium dictates the dispersion of the NPs within the BCP, which may render the NP compatible with either of the microphase-separated domains [40,41,42], or with the interface [35,36]. Furthermore, NPs may be chemically incompatible with the BCP matrix, which triggers the phase separation between NP-rich and BCP-rich domains [43].

The BCP ordered mesophase can be found to template the position of NPs, which may result in ordered NPs structures [44,45,46] such as arrays of NPs confined within lamellar domains [47]. Neutral NPs have been shown to change the orientation of lamellar BCP in thin films from parallel to perpendicular [48]. The conductivity of a BCP nanocomposite containing fillers was shown theoretically to depend strongly on BCP morphology [49]. Furthermore, the addition of colloids into a lamellar-forming BCP was experimentally shown to decrease the electric field threshold required to induce alignment [14].

Despite the varied uses of BCP melts as templates to control the position of colloidal NPs, BCP melts do not act as mere passive templates in the presence of NPs. Experiments and simulations have pointed out that the BCP may modify its morphology when a significant concentration of NPs is added into the system [50,51,52,53,54,55,56,57]. This phase transition is driven by the changes in the effective composition of the BCP upon the addition of selective NPs that effectively increase the overall composition of the hosting phase. Additionally, NPs can stabilise an otherwise metastable BCP phase, such as the case of hexagonally-perforated lamellar [58]. These results indicate that the formation of structures and ordering of BCP nanocomposites is a coassembly one, where the overall phase behaviour of the system depends both on the purely polymeric and colloidal properties. Additionally, NP size was shown to play a role in the localisation of NPs within the BCP domains [59,60,61,62]. The convergence of several length scales with comparable dimensions in BCP nanocomposites has been pointed out as a source of complexity [63]. For this reason, computer simulations have been extremely helpful to gain insight over the dynamics and phase behaviour of nanocomposite systems.

A variety of simulation methods have been used to study systems comprising BCP and NPs. These range from microscopic-scale simulations, such as molecular dynamics (MD) [64] and Monte Carlo (MC) [49,50,65,66], to theoretical approaches in strong segregation [67,68]. In order to study the equilibrium phase behaviour of BCP nanoscomposite systems, self-consistent field theory (SCFT) has been widely used [61,69,70,71].

In this work, we review recent TDGL models coupled with BD. In the 1980s, several authors derived the free energy functional of a BCP melt in terms of the local differential concentration of the BCP [72,73,74]. Together with the classical Cahn–Hilliard [75,76,77,78] description of the dynamics of phase-separating binary mixtures [79], this approach can be mapped [80] to the cell dynamic simulation (CDS) numerical scheme. In the 2000s, this scheme was successfully applied to a variety of BCP systems, describing their phase behaviour in equilibrium [81], under directed self-assembly [82,83,84] phase transition in the presence of external fields [85,86] and under confinement [87,88]. The versatility of the TDGL approach prompted the extension to hybrid models, including NPs [89]. This hybrid method, presented by Ginzburg et al. [90], combined the in-grid TDGL description of the BCP field as well as the out-of-grid BD for the NPs. It was applied to study the location of NPs within the BCP mesophase [90] and its effect on the dynamics of phase-separating mixtures [91]. More recently, TDGL/BD models have been extended to out-of-equilibrium systems to study BCP nanocomposites under electric fields [92,93]. In this work, we review recent applications of TDGL/BD models in equilibrium.

As is shown in detail in Section 2, TDGL models are highly coarse-grained and ignore several of the microscopic degrees of freedom of the polymeric system. This assures that the model is mesoscopic and, while this limits the applicability to the microscopic scale, it also allows to study collective behaviour and capture BCP properties occurring over larger time and length scales. In contrast, more sophisticated models such as MD, MC and SCFT have been used for rather microscopic properties such as the polymer radius of gyration in a BCP melt.

A widely used mesoscopic alternative approach to TDGL/BD is dissipative particle dynamics (DPD), which is entirely particle-based and performs coarse graining of a collection of molecules into mesoscopic beads following Newtonian dynamics with conservative, dissipative and random forces [94]. DPD has been extensively used in simulations of BCP and BCP nanocomposites [95,96,97,98]. In contrast with the TDGL/BD approach, DPD is not hybrid, and forces are generally derived from pairwise additive soft potentials.

### Complex Anisotropic NPs within BCP

Anisotropic NPs can self-assemble into a rich phase behaviour [99], ranging from liquid crystals to nanocrystals [100]. Furthermore, anisotropic NPs such as nanorods (NRs) have interesting applications based on their nanoscale behaviour, such as controlling the optical properties of the material [101] and applications for photovoltaic devices [102]. For these purposes, a precise control over their nanoscale positioning and orientation is desirable [5,103,104,105].

While BCP melts have been widely used to control the localisation of NPs within the mesophase, NPs with additional orientational degrees of freedom may be aligned with respect to the BCP domains. These orientational degrees of freedom may originate from NP anisotropic shape or chemically inhomogeneous coating along the surface of the NP. Given the inherent properties of symmetry of the BCP, segregating anisotropic NPs within the BCP melt can provide additional control in order to achieve a highly ordered material with precise control not only of the NP placement, but its orientation as well. A recurrent realisation of this is the alignment of NRs within lamellar or cylindrical BCP domains: relatively long NRs are softly confined within one the BCP phases and undergoes alignment sharing the BCP domain axis [106,107,108,109,110]. Conversely, NRs have been shown to template or seed the orientation of the BCP structure [111,112].

**Figure 1:**
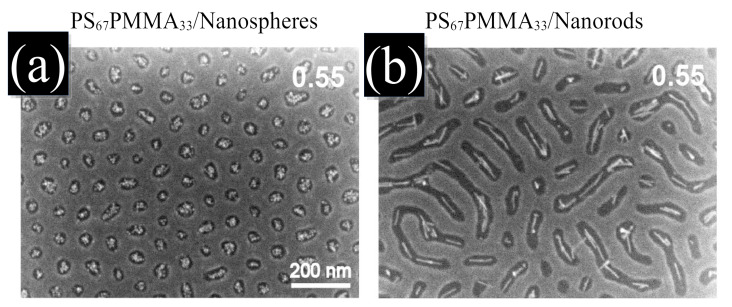
High-resolution SEM images of the coassembly of asymmetric PS-*b*-PMMA thin film with two NP shapes: (**a**) nanospheres and (**b**) NRs, with the same volume fraction of NPs $\phi_{p}=0.3$. NPs are compatibilised with the PS domains. The scale bar is the same in both images. Adapted with permission from Ref. [56]. Copyright 2014 American Chemical Society.

Experimentally, shorter NRs relative to the lamellar spacing have been mixed with BCP melts by Shenhar et al. to produce structures with colloidal ordering [113,114,115]. Figure 1 (right) shows the alignment of CdS NRs within the interior of PS domains [56]. Additionally, semiconductive NRs were found to organise side-by-side when compatibilised with one of the BCP phases in a lamellar morphology. In contrast to relatively long NRs, they oriented perpendicular to the lamellar domain axis, exposing the NR ends into the interface. Recently, nanoplates have been mixed with lamellar-forming BCP melts, again allowing to control not only the location of the NP but also the orientation of the NP with respect to the BCP mesophase [116].

The richness in the coassembly of BCP and anisotropic NPs has called for the use of theoretical and simulation methods to gain insight over their behaviour. Mesoscopic models such as DPD have been widely used. DPD models have been frequently used thanks to the simplicity to construct anisotropic NRs within the simulation method [117,118,119,120]. Furthermore, weak and strong segregation limit theory has been used to study the distribution and orientation of anisotropic NPs within the microphase-separated BCP [121,122,123].

In addition to shape anisotropy, chemically inhomogeneous NPs have attracted considerable attention in recent decades [124,125,126,127,128,129,130] due to their ability to self-assemble into complex structures. In particular, Janus nanoparticles (JNPs) have two chemically distinct halves (or hemispheres). They exhibit a rich phase behaviour when dispersed in solution [131,132,133,134,135], while their behaviour as amphiphillic-like particles has been exploited in ternary mixtures of JNPs and homopolymer blends [136,137]. These experimental works, along with DPD simulations [138,139,140], have concluded the slowed-down domain growth of the binary mixture when JNPs are present in the system. Recently, experiments by Yang et al. [141] were performed segregating JNPs within BCP interfaces. JNPs in BCP melts have been shown to posses a higher interfacial adsorption energy compared with evenly coated NPs [142].

In this review, we first present the hybrid TDGL model coupled with BD in Section 2. Here, both the basic model and the extensions for complex particles are introduced. Secondly, Section 3 describes recent results involving hybrid TDGL/BD simulations, again covering both isotropic and complex NPs. We conclude with a discussion in Section 4 as an overview of key elements of TDGL/BD in contrast with alternative methods.

## 2. Time-Dependent Ginzburg–Landau Coupled with Brownian Dynamics

**Figure 2:**
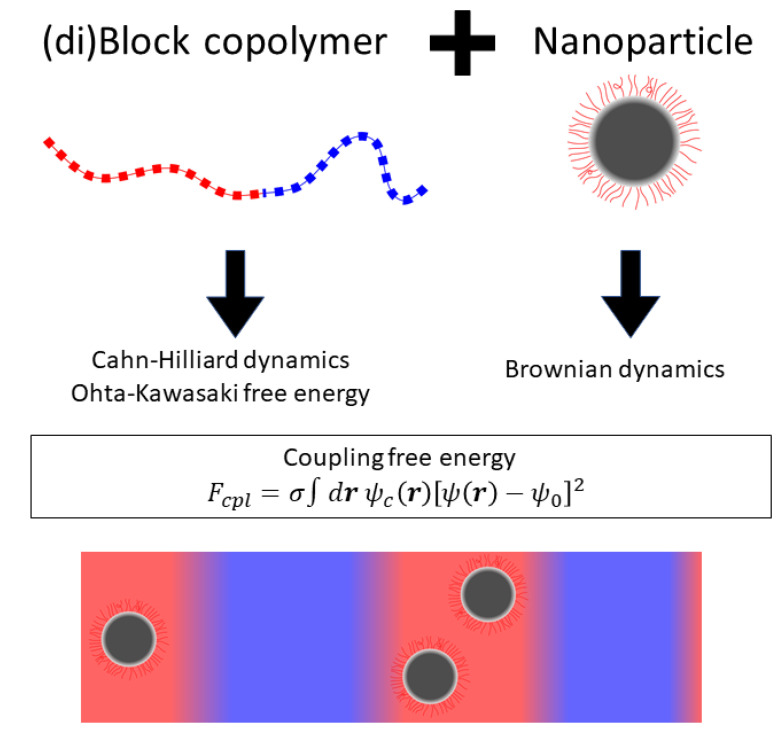
Schematic summary of the hybrid model described in Section 2.

In this section, we present a hybrid TDGL/Brownian dynamics model that was used to study a variety of systems involving BCP melts in the presence of NPs [143,144], firstly introducing the basic model followed by additions for systems involving anisotropic NPs. This approach combines a continuous in-grid description of the BCP melt following Cahn–Hilliard dynamics and a GL free energy, along with an individual description of the NP following Brownian dynamics. The two descriptions are coupled by an interaction term in the free energy, as shown graphically in the schematic summary in Figure 2.

The total excess coarse-grained free energy of a system comprising a BCP melt and a colloidal suspension of NPs can be decomposed into three contributions,

(1) $$F_{tot}=F_{pol}+F_{cc}+F_{cpl}$$

which correspond to the free-energy contributions of the polymer, colloid–colloid and polymer–colloid coupling, respectively. In this section, we specify the appropriate expressions for the free energy, as well as the dynamic equations for the BCP melt and the NPs. Afterwards, we present the extensions of the model to simulate complex NPs: firstly, nonspherical NPs and secondly, chemically inhomogeneous NPs (specifically, particles with chemically different hemispheres or JNPs).

### 2.1. Block Copolymer Dynamics: Time-Dependent Ginzburg–Landau

The diblock copolymer is characterized by the order parameter ψ(r,t) which represents the difference in the local volume fraction for the monomer A and B

(2) $$\psi \left(r,t\right)=\phi_{A}\left(r,t\right)-\phi_{B}\left(r,t\right)+\left(1-2f_{0}\right)$$

with respect to the relative volume fraction of A monomers in the diblock, $f_{0}=N_{A}/\left(N_{A}+N_{B}\right)$.

The order parameter must follow the continuity equation in order to satisfy the polymer mass conservation (strictly speaking, only outside of a NP; see Section 2.3 for a discussion on boundary conditions)

(3) $$\frac{\partial \psi (r,t)}{\partial t}=-\nabla \cdot j\left(r,t\right)$$

If the polymer relaxes diffusely towards equilibrium, the order parameter flux can be expressed in the form

(4) j(r,t)=−M∇μ(r,t)

as a linear function of the chemical potential

(5) $$\mu \left(r,t\right)=\frac{\delta F_{tot}\left[\psi \right]}{\delta \psi }$$

Introducing these equations into the continuity equation, and taking into account the thermal fluctuations, we obtain the Cahn–Hilliard–Cook Equation (CHC) [75,76,77,78]

(6) $$\frac{\partial \psi (r,t)}{\partial t}=M\;\nabla^{2}\left[\frac{\delta F_{tot}\left[\psi \right]}{\delta \psi }\right]+\xi \left(r,t\right)$$

where *M* is a phenomenological mobility constant and ξ is a white Gaussian random noise which satisfies the fluctuation–dissipation theorem [145].

The copolymer free energy is a functional of the local order parameter, which can be expressed in terms of the thermal energy $k_{B}T$ as [72]

(7) $$F_{pol}\left[\psi \left(r\right)\right]=\int dr\left[H\left(\psi \right)+\frac{1}{2}D{\left|\nabla \psi \right|}^{2}\right]+\frac{1}{2}B\int dr\int dr^{\prime }\;G\left(r-r^{\prime }\right)\psi \left(r\right)\psi \left(r^{\prime }\right)$$

where the first and second terms correspond to the short and the long-range free energy contributions, respectively. The coefficient *D* is a positive constant that accounts for the cost of polymer concentration inhomogeneities. The Green function $G(r-r^{\prime })$ for the Laplace Equation satisfies $\nabla^{2}G\left(r-r^{\prime }\right)=-\delta \left(r-r^{\prime }\right)$, and *B* is a parameter that introduces a chain-length dependence to the free energy [72,146]. This long-range contribution to the free energy accounts for the connectivity of the BCP chain.

The local contribution to the free energy is specified with H(ψ) [146,147] which drives the phase separation,

(8) $$H\left(\psi \right)=\frac{1}{2}\left[-\tau_{0}+A{\left(1-2f_{0}\right)}^{2}\right]\psi^{2}+\frac{1}{3}vs.\left(1-2f_{0}\right)\psi^{3}+\frac{1}{4}\psi^{4}$$

where $\tau_{0},A,v,u$ are phenomenological parameters [81] which can be related to the BCP molecular specificity. Previous works [72,81,143] describe the connection of these effective parameters to the BCP molecular composition. $\tau^{\prime }=-\tau_{0}+A{\left(1-2f_{0}\right)}^{2}$, *D* and *B* can be expressed [72] in terms of degree of polymerization *N*, the segment length *b* and the Flory–Huggins parameter χ(inversely proportional to temperature). As previously shown [148,149], TDGL can be used along with more detailed approaches such as dynamic self-consistent field theory (DSCFT), using TDGL as a precursor in exploring parameter space due to the computationally inexpensiveness nature of TDGL.

### 2.2. Numerical Scheme: Cell Dynamic Simulations

The CDS method can be used as a discretisation of the partial differential Equation (6). This method, developed by Oono and coworkers [150,151,152,153], displayed a high stability while using large time steps and has been extensively used for phase separation of binary mixtures and BCP systems [154].

In CDS, the Laplacian is highly isotropic and is expressed in terms of the local average operator 〈〈*〉〉 as $\nabla^{2}X=\frac{1}{{\left(\delta x\right)}^{2}}\left[\langle \langle X\rangle \rangle -X\right]$ where δx is the grid spacing. In three dimensions, the CDS method employs a discretised Laplacian using a 27-point stencil.

(9) $$\langle \langle \psi \rangle \rangle =\frac{6}{80}\sum_{NN}\psi +\frac{3}{80}\sum_{NNN}\psi +\frac{1}{80}\sum_{NNNN}\psi$$

with *NN*, *NNN* and *NNNN* meaning nearest neighbours, next-nearest neighbours and next-next-nearest neighbours, respectively. In 2D, instead, CDS uses a 9-point average operator as

(10) $$\langle \langle \psi \rangle \rangle =\frac{1}{6}\sum_{NN}\psi +\frac{1}{12}\sum_{NNN}\psi$$

Alternative discrete Laplacians have been studied in the context of the Cahn–Hilliard equation, which determined its stability and isotropy properties [80].

### 2.3. Coupling BCP Field and NPs

Contrary to the BCP melt—described continuously by differences in concentration—colloidal NPs are described individually. The presence of a number $N_{p}$ of colloidal NPs immersed in the BCP melt is introduced by the coupling term in the total free energy, Equation (1), which is expressed as

(11) $$F_{cpl}=\sum_{i=1}^{N_{p}}\sigma \int dr\psi_{c}\left(r\right){\left[\psi \left(r\right)-\psi_{0}\right]}^{2}$$

where the overall strength of the polymer–particle coupling is set by parameter σ.

**Figure 3:**
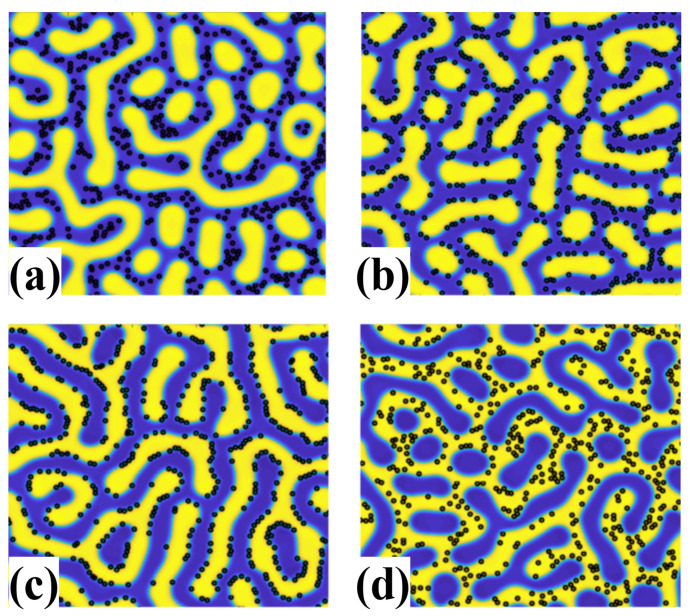
Role of affinity parameter $\psi_{0}$ controlling the dispersion of a low concentration $\phi_{p}=0.06$ of NP within a lamellar-forming BCP melt. NPs (shown in black) are segregated within the blue phase in (**a**) for $\psi_{0}=-1$, slightly off-center within the interface in (**b**) for $\psi_{0}=-0.33$, symmetrically at the interface in (**c**) for $\psi_{0}=0$ and within the yellow phase in (**d**) for $\psi_{0}=0.67$. Reprinted with permissions from Ref. [57]. Copyright 2018 Wiley.

The role of $\psi_{0}$ is to specify the chemical affinity of the NP with each part of the BCP melt and can be related to the coating of NP surface with grafted polymers to control the segregation of NPs within the microphase-separated BCP domains. Figure 3 shows the role of $\psi_{0}$ at controlling the positioning of NPs within a lamellar-forming BCP, which can result in the segregation of NPs within either BCP phase, or within the interface.

The size and shape, as well as the distinction between the interior and exterior of the particle, is introduced by the undeformable tagged function $\psi_{c}$, which moves along the center of mass of the particle and depends, for isotropic particles, on the distance $r=|r_{i}-r|$ between the NP centre of mass $r_{i}$ and a point in the BCP field r,

(12) $$\psi_{c}\left(r\right)=\exp\left[1-\frac{1}{1-{\left(r/R_{eff}\right)}^{\alpha }}\right]$$

which takes a value $\psi_{c}\left(r>R_{eff}\right)=0$ for distances larger than the cut-off, i.e., the effective radius of the NP $R_{eff}$, which sets the range of the coupling interaction. The smoothness of the decay is controlled by parameter α, which allows to define a hard-core radius $R_{0}=R_{eff}/{\left(1+1/\ln2\right)}^{1/\alpha }$ as the distance at which the tagged function $\psi_{c}\left(R_{0}\right)=1/2$ has decayed half its value at the center. A value α=2 was selected to ensure a smooth decay [143]. Alternative choices of the tagged function have included hyperbolic tangents [155], which lack the compact functional form of Equation (12), with a vanishing derivative at the edge.

We note that in Equation (11), the BCP field ψ(r) is described continuously and numerically evaluated in a grid, as shown in Section 2.2. Meanwhile, the colloidal NPs move as individual particles and are numerically evaluated outside of the grid. This constitutes the model as a hybrid in-grid/out-of-grid scheme where both descriptions are coupled via Equation (11). In particular, the coupling free energy is evaluated as an integral within the grid. The tagged field ψ(r) acts as the mediator between the in-grid and the out-of-grid description, as the distance $r=|r_{i}-r|$ relates the position of the NP $r_{i}$ defined continuously (i.e., floating point), with the space point r being a discrete point in the grid (i.e., integer). We note that this introduces a lower bound to the range of possible values of $R_{eff}⪆1$, which should be roughly larger than one grid.

In the presence of NPs, the free energy of the BCP needs to account for the presence of NPs in Equation (7). In principle, the presence of NPs could be introduced via boundary conditions at the surface of the NP, imposed on the BCP field ψ and on the BCP flux in Equation (4). Computationally, however, this presents difficulties for calculating off-grid boundaries for each NP at each iteration. An explicit treatment of the boundary conditions has been used in 3D systems with spherical symmetry [156,157], which can be related to the case of a single immobile NP. An alternative approach, used in this work, is to allow the BCP field ψ to be defined within the NP interior, as is clear in Equation (11). This eliminates the need for explicit boundary conditions on ψ but requires a slight modification of the long-range free energy term in Equation (7), that is, the double integral term, as

(13) $$F_{pol}^{lr}=\frac{1}{2}B\int dr\int dr^{\prime }\;G\left(r-r^{\prime }\right)\psi \left(r\right)\psi \left(r^{\prime }\right)\left[1-P(r^{\prime })\right]$$

where a continuous field P(r) takes value P(r)=1 inside a NP hard-core radius and 0 otherwise. Regions of system space where *P* is nonzero indicate that the polymer field ψ represents a homopolymer mixture, rather than a BCP melt. This is equivalent to assuming that the BCP field outside of the NP core interacts with the interior as if the NP interior was a homopolymer mixture. Along these lines, we can interpret the modified long-range free energy term in Equation (13) as an interaction term between the BCP field at point r, ψ(r), and the BCP field at another point $r^{\prime }$, $\left[1-P\left(r^{\prime }\right)\right]\psi \left(r^{\prime }\right)$, mediated by the Green function. We note that r and $r^{\prime }$ are dummy variables and interchangeable.

**Figure 4:**
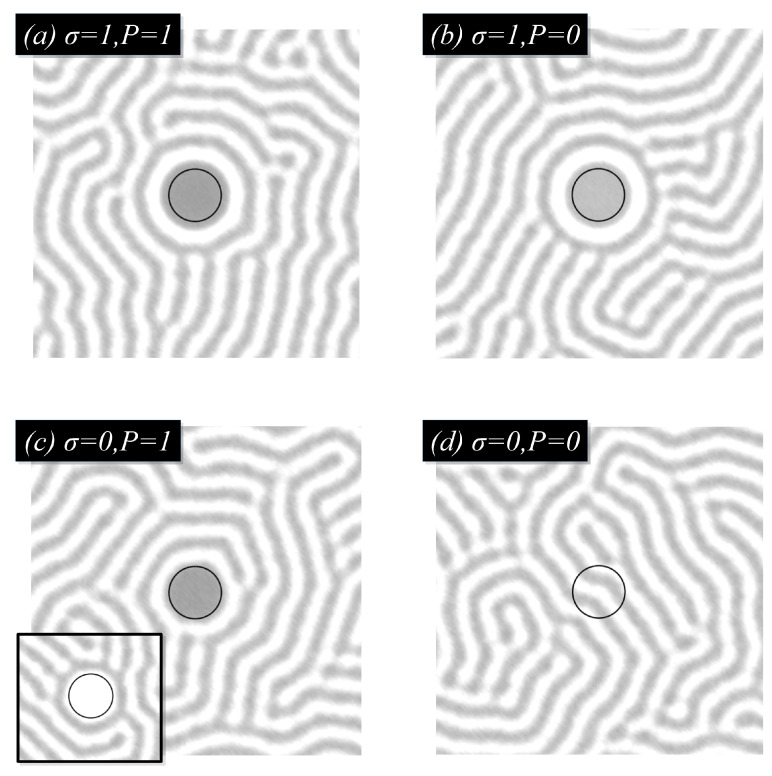
Single selective particle $\psi_{0}=-1$ within a lamellar-forming BCP ($f_{0}=1/2$) under various conditions for the particle–polymer interaction.

The role of P(r) becomes clear in Figure 4, where a relatively large NP is mixed with a lamellar-forming BCP with affinity $\psi_{0}=-1$ towards the grey phase. If the coupling free energy is introduced with σ=1 in (a) and (b), P(r) plays a secondary role, as the value of ψ(r) is mostly dominated by the minimisation of the coupling free energy. However, if σ is negligible, we can observe that the polymer field within the NP depends strongly on P(r): in (c) with P=1 inside of the NP, the polymer field evolves as a binary mixture until a single-phase is achieved (gray phase in figure, white phase in inset); contrary to that, if P=0 inside of the particle, the NP is completely noninteracting with the BCP.

Introducing the P(r) field in the long-range interaction allows to better capture the expected boundary conditions in the particle–polymer interface, even though they are not explicitly present in the system. An explicit treatment of the particle–polymer boundary conditions by Pinna et al. [156,157] clearly indicates that neutral NPs, $\psi_{0}=0$, produce perpendicular alignment of lamellar domains in the particle–polymer interface. On the other hand, selective NPs result in parallel alignment.

The coupling free energy shown in Equations (11) and (12) describes a spherical NP with homogeneous coating throughout its surface. Extensions of this simple model are shown in Section 2.5 for nonspherical particles and Section 2.7 for chemically inhomogeneous NPs.

### 2.4. Colloidal Dynamics: Brownian Motion

Colloids undergo diffusive dynamics, described by the Langevin equation in the over-damped regime. The center of mass of each colloid $r_{i}$ is considered to follow BD, that is,

(14) $$\frac{dr_{i}}{dt}=\frac{1}{\gamma }\left(F_{i}^{cc}+F_{i}^{cpl}+\sqrt{2k_{B}T\gamma }\xi_{i}\right)$$

with γ as the friction coefficient, $k_{B}T$ as the NP thermal energy and $\xi_{i}$ as a random Gaussian term with zero mean $\langle \xi_{i}\left(t\right)\rangle =0$ and with amplitude satisfying fluctuation dissipation theorem $\langle \xi_{j}\left(t^{\prime }\right)\xi_{i}\left(t\right)\rangle =\delta_{ij}\delta \left(t-t^{\prime }\right)$. The coupling force $F_{i}^{cpl}=-\nabla F_{cpl}$ accounts for the interaction between the NPs and the BCP medium. Similarly, NPs experience forces due to the colloid–colloid interactions $F_{i}^{cc}=-\nabla F_{cc}$, with

(15) $$F_{cc}=\sum_{ij}U\left(r_{ij}\right)$$

and $U(r_{ij})$ being a pairwise additive potential that depends on the centre of mass distance between particles. A variety of intercolloidal potentials have been used in the literature of colloidal suspension BD simulations.

In order to introduce repulsive interactions that prevent overlapping between NPs, a Yukawa-like potential was introduced as

(16) $$U\left(r\right)=U_{0}\left[\frac{\exp(1-r/R_{12})}{r/R_{12}}-1\right]$$

where $R_{12}$ sets the cut-off interaction distance for the particle–particle potential and $U_{0}$ specifies the strength of the interparticle potential. $U_{0}$ can be specified with respect to the thermal energy $k_{B}T$ and the coupling interaction to prevent overlapping between particles.

### 2.5. Anisotropic Colloids: Nonspherical Particles

**Figure 5:**
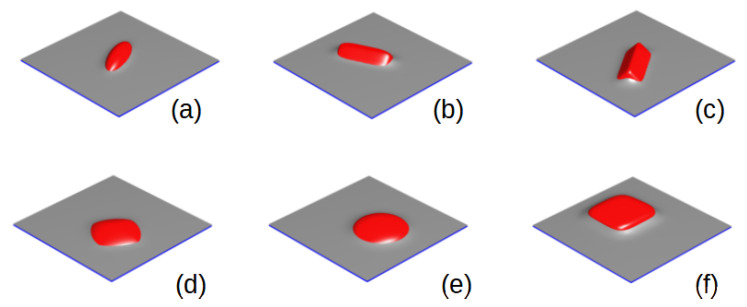
Assembly at a flat interface of neutral NPs with various shapes controlled by the super-ellipse parameter. The parameters are grouped as (a,b,c,n) for the three semiaxis and the exponent *n*, respectively: (**a**) (8,3,3,1); (**b**) (8,3,3,2); (**c**) (8,3,3,4); (**d**) (3,8,8,0.75); (**e**) (3,8,8,1) and (**f**) (3,8,8,2).

In order to simulate NPs with nonspherical shapes, it is clear that two elements need to be modified in the free-energy description of the system: the polymer–particle coupling needs to account for the anisotropic shape of the particles, and the particle–particle interaction should reflect the orientational dependence that arised from the nonspherical shape of the particles. The shape of the particle with regards to the particle–polymer interaction can be tuned by modifying the tagged function described in Equation (12). In order to respect the decay profile of the tagged function, the following functional expression is chosen:

(17) $$\psi_{c}\left(s\right)=\exp\left[1-\frac{1}{1-s(r)}\right]$$

where the generalised metric function *s* can be identified with $s={\left(r/R_{eff}\right)}^{\alpha }$ to recover the isotropic shape of the particle. For a nonspherical NP, the shape of the particle can be generalised to be represented by the family of curves known as hyper-ellipses (super-ellipses in 2D), described by the expression

(18) $$s\left(r\right)={\left[{\left|\frac{x^{\prime }}{a}\right|}^{2n}+{\left|\frac{y^{\prime }}{b}\right|}^{2n}+{\left|\frac{z^{\prime }}{c}\right|}^{2n}\right]}^{1/n}$$

where the exponent 1/n rescales the decay [158] of $\psi_{c}$ to assure the smooth profile of the coupling interaction. Coordinates $x^{\prime }$, $y^{\prime }$ and $z^{\prime }$ are body-fixed along the three main axis *a*, *b* and *c* of the anisotropic NP, respectively. We note that if $a=b=c=R_{eff}$, we recover the tagged function expression in Equation (12). The body-fixed coordinates $x_{i}^{\prime }$ (i = x,y,z) can be related to the system-fixed $x_{i}$ via the rotation matrix [159] $x_{i}^{\prime }=R_{ij}\left(\alpha ,\beta ,\gamma \right)x_{j}^{\prime }$, with $R_{ij}\left(\alpha ,\beta ,\gamma \right)$ being the 3D rotation matrix given by the three Euler angles α,β and γ that characterise the state of rotation of the particle. Equation (18) characterises a 3D anisotopic NP with three main semiaxis a≥b≥c and exponent *n*, which specifies the subfamily of hyper-ellipses: for n<1/2 for starlike particles, n=1/2 for 3D rhomboids, n=1 for ellipsoids and n>1 for increasingly sharper cuboid-shaped particles. Figure 5 shows several 3D NP shapes for neutral NPs ($\psi_{0}=0$) anchoring at an interface (shown in grey). The variety of NP shapes (see caption for aspect ratios) are found segregated within the interface, and aligned along it, in order to minimise the coupling free energy, Equation (11).

**Figure 6:**
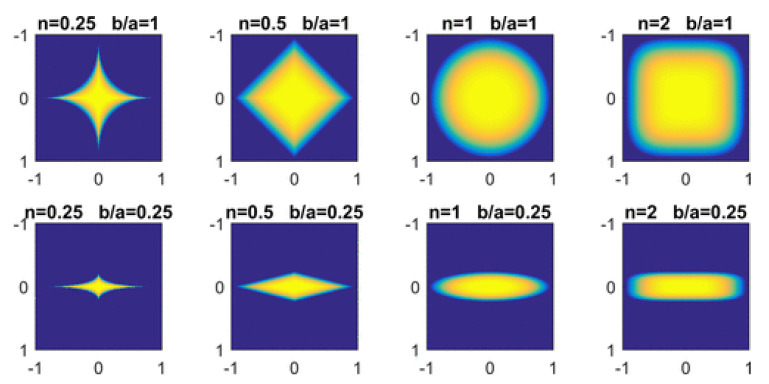
Colour map of $\psi_{c}$ as described in Equations (12) and (18) for several values of *n* and b/a. The particles are assumed to have no rotation ϕ=0 and larger semiaxis a=1. Reprinted with permission from Ref. [160] Copyright 2019 American Chemical Society.

In 2D ($z^{\prime }=0$), the orientation of an anisotropic NP is specified with a single orientational degree of freedom ϕ. Furthermore, an aspect ratio e=b/a can be defined, which distinguishes, for example, between circular (e=1) and ellipsoidal (e<1) NPs, as in the top and bottom row of Figure 6. More generally, each rotated anisotropic colloid is characterised by a unit vector ${\hat{u}}_{i}=\left(\cos\phi_{i},\sin\phi_{i}\right)$ which depends on an angle $\phi_{i}$. The simplicity of the coupling free energy allows us to simulate a vast range of NP shapes. The family of superellipses in two dimensions includes ellipses, rectangles, rhombus and starlike particles. Many of these shapes can serve as a faithful representation of experimentally-motivated nonspherical colloids such as nanocubes, nanospheres or NRs.

An alternative approach to simulate anisotropic NPs in the context of TDGL/BD models has been presented using a double integral that spans the surface (3D system) or line contour (2D systems) of a NP [161,162,163,164]. In contrast with Equation (11), in this method, the NP shape is introduced explicitly by integration of the particle–polymer interaction, with a given length that characterises the range of the interaction. In practice, both approaches result in a similar scheme for the particle–polymer interaction. The model described in this work (Equation (11)) simplifies the particle–polymer interaction by considering a single integration that spans both the particle–polymer surface (contour in 2D), as well the interior volume (surface in 2D). This is advantageous from a computational point of view and simplifies the generalisation into arbitrary shapes, as shown in Equations (18). On the other hand, the coupling interaction proposed by Balazs et al. [161] is closer to experimental NP-polymer interfaces where the interaction takes place at the exterior (corona) of the NP. We note that in simulations, the interior of the NP adds a negligible contribution to the free energy, as the polymer field is approximately equal to the NP affinity $\psi \left(r\right)\approx \psi_{0}$ at the centre of the particle.

### 2.6. Anisotropic Potentials

Both in 2D and 3D, an appropriate pair-wise additive potential needs to be introduced in order to properly simulate anisotropic NPs, substituting the isotropic Yukawa-like potential described in Equation (16). The particle–particle potential needs to capture the nonsherical shape of the particle when two colloids interact. In the literature on coarse-grained simulations of anisotropic NPs, a wide variety of anisotropic potentials have been introduced. The Gay–Berne potential [165,166] has been widely used in simulations of liquid crystals and is derived from the Gaussian overlap between ellipsoids [167,168]. The interparticle potential is written as

(19) $$U_{GB}\left({\hat{u}}_{1},{\hat{u}}_{2},r\right)=\epsilon \left({\hat{u}}_{1},{\hat{u}}_{2},\hat{r}\right)\left[{\left(\frac{1}{r-\sigma ({\hat{u}}_{1},{\hat{u}}_{2},r)}\right)}^{12}-{\left(\frac{1}{r-\sigma ({\hat{u}}_{1},{\hat{u}}_{2},r)}\right)}^{6}\right]$$

which can be shown to be a modified Lennard–Jones potential with anisotropic length and energy scales, respectively, $\sigma ({\hat{u}}_{1},{\hat{u}}_{2},r)$ and $\epsilon ({\hat{u}}_{1},{\hat{u}}_{2},\hat{r})$. The orientation of two interacting ellipsoids is given by the unit vectors ${\hat{u}}_{i}$, and the interparticle unit vector is $\hat{r}$. The Gay-Berne potential is applicable to ellipsoids corresponding to NPs with exponent n=1, as shown in Equation (18). In Figure 6, they correspond to the third column.

Even though the Gay–Berne potential is considerably standard in molecular dynamic simulations of ellipsoids, it requires a relatively small time step when compared with the Yukawa-like potential shown in Equation (16). This limits the CDS ability to use large time steps and could potentially act as a bottleneck for the computational time. For this reason, a modified Yukawa potential that incorporates the angle-dependent length scale $\sigma ({\hat{u}}_{1},{\hat{u}}_{2},r)$ could be used, which has the same functional form as the standard Yukawa-like potential, but with the orientational dependence introduced implicitly via the particle–particle diameter

(20) $$U\left(r\right)=U_{0}\left[\frac{\exp(1-r/\sigma \left({\hat{u}}_{1},{\hat{u}}_{2},r\right))}{r/\sigma ({\hat{u}}_{1},{\hat{u}}_{2},r)}-1\right]$$

which allows to preserve a relatively large time step while capturing the anisotropic shape of a prolate ellipsoid.

Contrary to ellipsoids, there is no general potential that is applicable to particles of arbitrary shape belonging to the family of super-ellipses. In order to simulate rectangular and rhomboidal-shaped particles, a completely repulsive potential, which is proportional to the overlapping area between two arbitrary-shaped rhomboids, was introduced in reference [160],

(21) $$U_{fitted}\approx U_{0}A_{overlap}\left({\hat{u}}_{1},{\hat{u}}_{2},r\right)$$

which can capture, for example, the 4-fold symmetry in the interaction between 2D squares. This potential captures the NP shape and is limited to instances of overlapping, which are determined exactly via the separation axis theorem [169], which states that the overlapping of two convex objects in 2D is prevented if a line can be drawn separating both objects. If overlapping occurs, the potential that particle pairs experience is fitted to capture the shape of the particles. Forces and torques are derived from this fitted potential. The overall scale of the potential is tuned to prevent overlapping.

Other works [161] performed simulations of NRs as one-dimensional NPs, lacking any width. This simplifies the treatment of the particle–particle interaction while maintaining the basic feature promoting nematic alignment between NRs. However, this approach is not able to capture the minor dimension of the NR, which may be relevant at high local concentrations [113,114,115,170].

### 2.7. Anisotropic Colloids: Chemically Inhomogeneous Coatings

The affinity parameter introduced in the coupling free-energy term in Equation (11) characterises the wetting of the NP towards the BCP field ψ(r). In order to model the inhomogeneous chemical properties of Janus NPs, the affinity parameter $\psi_{0}\left(\phi_{i}\right)$ can be split into two values for each side of the NP [162]. By doing so, the coating of each side of the NP can be considered different, with distinct affinity values $\psi_{+}$ and $\psi_{-}$ characterising the wetting of each half towards the BCP medium [171].

By *breaking-up* the affinity of the particle $\psi_{0}\left(\phi \right)$, the explicit dependence on the particle’s orientation results in a torque acting on the orientational degree of freedom ϕ. The dynamics of ϕ are diffusive, as is described in Section 2.8 (see Equation (23)).

The degree of inhomogeneity and mean affinity can be characterised by the parameters

(22) $$\Delta \psi_{0}=\psi_{+}-\psi_{-},{\bar{\psi }}_{0}=\frac{1}{2}\left(\psi_{+}+\psi_{-}\right)$$

which allows to draw a correspondence of a JNP with its chemically homogeneous counterpart by setting $\Delta \psi_{0}=0$ for a given mean affinity ${\bar{\psi }}_{0}$.

### 2.8. Extension for Dynamics of Orientational Degrees of Freedom

Nonspherical NPs described with Equation (18) and chemically inhomogenous NPs introduce an additional orientational degree of freedom $\phi_{i}$. In accordance with overdamped Langevin dynamics described in Equation (14), the orientational degree of freedom $\phi_{i}$ follows

(23) $$\frac{d\phi_{i}}{dt}=\frac{1}{\gamma_{R}}\left(M_{i}^{c-c}+M_{i}^{cpl}+\sqrt{2k_{B}T\gamma_{R}}\xi_{R}\right)$$

where $M_{i}^{c-c}$ and $M_{i}^{cpl}$ are, respectively, colloid–colloid and colloid–polymer torques. The coupling and intercolloidal torques can be calculated from the free energy of the system $M_{i}=-\partial F_{tot}/\partial \phi_{i}$. In this work, we do not introduce interparticle torques for chemically inhomogeneous particles, that is, $M_{i}^{cc}$ only results from the GB potential (Equation (19)) or the fitted repulsive potential (Equation (21)).

In 3D, three Euler angles, α,β and γ, are used to specify the orientation of nonspherical particles, with their dynamics similarly following overdamped Langevin dynamics in the presence of colloid–colloid and coupling torques, as discussed for the 2D case.

### 2.9. Parallelisation

The TDGL scheme presented in Section 2.1 and Section 2.2 is relatively fast compared with methods such as molecular dynamics or Monte Carlo, which resolve the microscopic state of the system. Nonetheless, the computational time requirement scales with the system size, which limits the ability to reach large system sizes. This is particularly relevant in BCP systems where small system sizes can pin the system into artificial intermediate states.

Parallelisation is a HCP technique commonly used to speedup computational tasks by dividing the total workload into several computer processors, which reduces the overall computational time. A spatial decomposition algorithm has been commonly used to allocate one processor for each subdivision of the whole system [172]. The lack of long-range calculations assures that interprocessor communications only need to be performed across immediate neighbors.

Commonly, the performance of a parallel code is tested by observing the reduction in the elapsed time for an increasingly larger number of processors. The strong scaling $S=T\left(1\right)/T\left(n_{p}\right)$ is defined as the elapsed time *T* using a single processor run over the elapsed time using $n_{p}$ processors. Ideally, this scaling increases linearly with the number of processors, i.e., $S=n_{p}$. In practice, several issues may affect the scalability of the parallel code (hardware limitations, communication between processors, etc.). A parallel algorithm using Coarray Fortran [173,174] was presented [175] where a satisfactory scaling was shown up to 4096 CPU processors in the absence of NPs. The overall system box was decomposed into smaller subsystems, which are assigned to an image (corresponding to a processor). In the Coarray parallelisation scheme, each image possess its own set of standard variables. On the other hand, an image can read the value of a variable of coarray type from another image. This, along with introducing ghost layers, allows to communicate between processors. The boundary conditions can be naturally introduced by specifying the ghost layer of the outer images.

**Figure 7:**
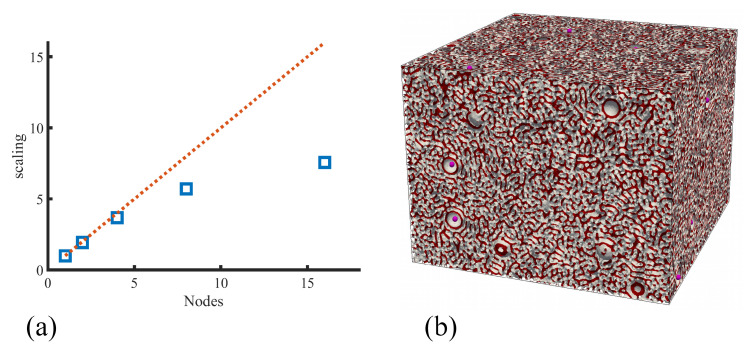
Performance of the parallel hybrid algorithm. In (**a**), strong scaling is shown for a system with $N_{p}=10^{4}$ particles in a $V=256^{3}$ box system, using 8 processors in the CSCS supercomputer. In (**b**), a snapshot of a relatively large system with $V=400^{2}\times 300$ with $N_{p}=100$ particles with radius $R_{eff}=13.3$ is shown. Reprinted with permission from Ref. [175] Copyright 2021 Wiley.

Additionally, a spatial decomposition approach was used for the dynamics of colloidal NPs, consistent with the one used for the polymer matrix. The main computational bottleneck at high concentrations was found to be the calculation of the polymer–particle coupling [175], far above the calculation of particle–particle forces. The strong scaling analysis shown in Figure 7a shows a close-to-ideal scaling up to 64 cores and a simulation snapshot of a considerably large system with V=400×400×300 grid points box size.

Other particle-based simulation methods such as DPD can achieve relatively large system sizes due to a combination for coarse graining and paralelisation (e.g., DL_MESO [176]). However, the calculation of interparticle forces often carries a heavy computational load. The parallel TDGL combined with BD allows to simulation hybrid in-grid/out-of-grid systems taking advantage of multicore (super)computers to reach large system sizes.

## 3. Applications of Hybrid TDGL/BD Models

In this section, we first review the main results obtained using TDGL/BD methods for simple isotropic NPs in 2D and 3D. Then, we review more complex setups of anisotropic NPs due to the nonspherical shapes and inhomogenous coatings.

### 3.1. Mixtures of BCP and Isotropic NPs

**Figure 8:**
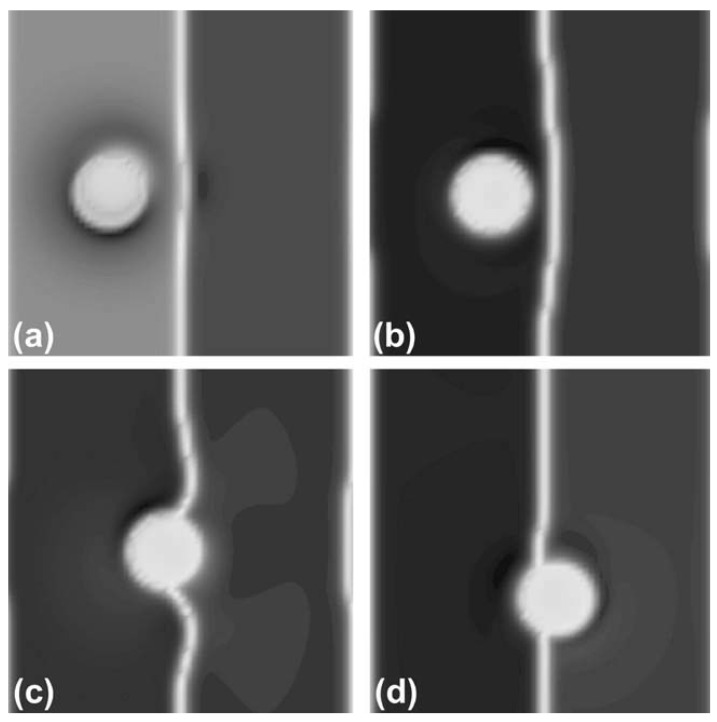
Almost-neutral ($\psi_{0}=0.2$, shown in white) particle adsorbing into a binary immiscible mixture for timesteps (**a**) 100, (**b**) 190,000, (**c**) 250,000 and (**d**) 500,000. Reprinted with permissions from Ref. [143], Copyright 2011 Wiley.

The hybrid TDGL/BD scheme with coupling free energy, as specified in Equation (11), was used to study the segregation of an almost-neutral NP into a binary mixture interface. This simplified setup allowed to study the time evolution of the NP adsorption into the interface by considering a particular limit of the BCP nanocomposite model [143]. By setting B=0 (see Equation (7)), it was possible to simulate an immiscible binary mixture as a particular case of the BCP melt, eliminating the long-range interaction term that accounts for the connectivity of the BCP chain. This regime may correspond to the phase separation of a binary homopolymer mixture. A relatively large colloidal NP with an affinity $\psi_{0}=0.2$ is preferentially segregated towards the interface of a binary mixture in Figure 8, as the colloidal particle experiences forces until the equilibrium contact angle at the interface is achieved [143]. The particle is initially placed away from the interface in (a), which introduces a distortion in the nearby polymer field (due to chemical potential contributions from Equation (11)) in (b) and (c), and eventually is placed at the equilibrium-decorating interface. Such an almost-neutral particle behaves in a similar way as a surfactant particle, as it segregates into the interfaces in order to alleviate the interfacial tension between two demixed phases. The placement of the colloid is not perfectly symmetric due to the nonzero value of the affinity parameter $\psi_{0}=0.2>0$. Experimentally, this can be mapped to a NP with a random graft of A and B polymer grafts, but slightly unequal, with a higher fraction of monomers of the positive kind ψ∼1.

**Figure 9:**
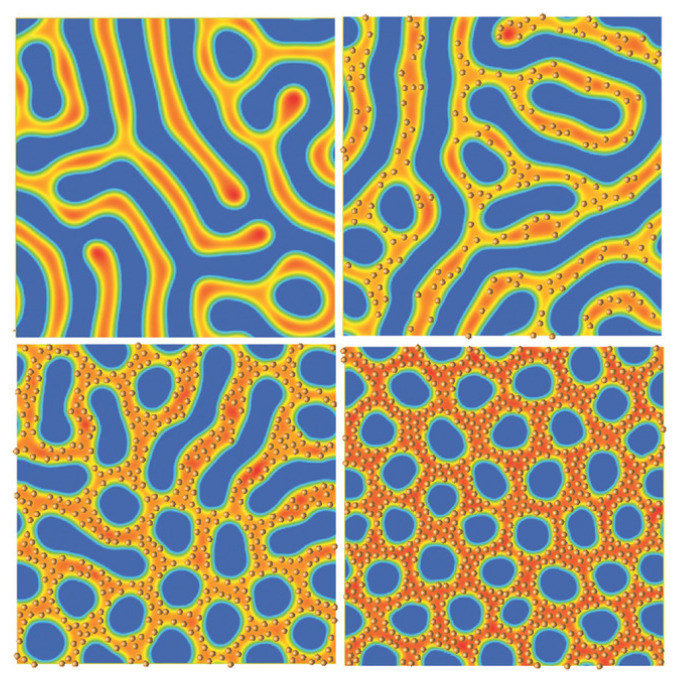
Phase transition lamella to ordered cylindrical induced by NPs as we increase their volume fraction with values $\phi_{p}=0$, 0.15, 0.35 and 0.55, respectively. Reprinted with permissions from Ref. [144], Copyright 2017 Wiley.

Contrary to neutral NPs, selective ones preferentially segregate within one of the BCP phases. This can be the consequence, for example, of the grafting of polymer chains to the NP surface. Within the hybrid TDGL/BD model, the preferential wetting can can be introduced by a coupling free-energy term that is minimised when a NP is placed within one of the bulk phases, i.e., $\psi_{0}\approx \pm 1$ in Equation (11). Selective NPs have been widely dispersed experimentally within BCPs to obtain precise control over their placement at the nanoscopic scale [10]. In Figure 9, selective NPs are dispersed within one of the BCP phases in a symmetric BCP in the strong segregation. In the absence of NPs, the BCP is lamellar-forming (top left). NPs (circles in brown) are clearly dispersed within the orange phase, where they experience Brownian motion. Upon the addition of NPs in the system, a clear transition in the BCP can be observed: In Figure 9, bottom left, we observe the appearance of more circular domains which are less connected, while in the bottom right the transition is completed. The initially lamellar-forming BCP acquires a circular domain morphology in the presence of a relatively high concentration $\phi_{p}=0.55$ of NPs.

The morphological phase transition reported in Figure 9 is the result of the increase in the effective composition of the hosting BCP phase, following the addition of a finite volume fraction of NPs $\phi_{p}$. NPs effectively increase (or decrease) the overall composition of the hosting domains (or the incompatible domains). Experiments largely reported these type of transitions involving isotropic NPs [51,52,55] and nonspherical NRs [53,56], in bulk or under confinement in thin films. Similarly, the addition of homopolymer chains into a BCP melt has been experimentally shown to induce an equivalent transition driven by the effective change in the overall BCP composition ratio [177,178]. These are all instances of order-to-order phase transition due to the effective change in the BCP composition ratio due to the introduction of additives (nanospheres, NRs or homopolymers).

**Figure 10:**
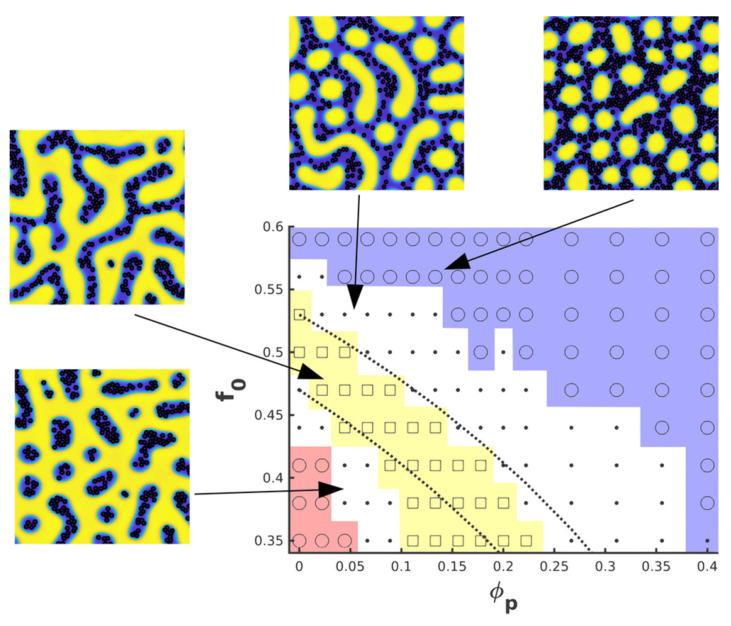
Morphology of a BCP/NP system with a $f_{0}$ ratio and $\phi_{p}$ NPs which are strongly compatible toward one of the phases. The BCP is in the strong segregation regime. Shown phases are circular phase (circles), mixed phase (dots) and lamellar (squares). Reprinted with permissions from Ref. [57], Copyright 2018 Wiley.

In order to quantify the effect of NPs in the BCP morphology, Huh et al. [50] introduced an effective BCP composition parameter $f_{eff}=\phi_{p}+\left(1-\phi_{p}\right)f_{0}$ to account for the changes in the overall composition of the BCP melt upon the addition of selective NPs. The phase diagram could be obtained using Monte Carlo simulation methods, where the presence of NPs was shown to shift the order–order phase transition. Figure 10 shows the corresponding phase diagram using a TDGL/BD model [57]. The flexibility of the model allows to also study the role of the NP affinity: neutral NPs were shown to segregate towards the interface (as shown in Figure 8) and, at high enough concentration, break the lamellar interface into smaller domains to promote the creation of longer interface length. A lamellar to bicontinuous transition in the BCP in the presence of neutral NPs has been reported theoretically [67,68,179], given by the vanishing of the bending modulus of the BCP, which is in accordance with experimental findings [35,180].

**Figure 11:**
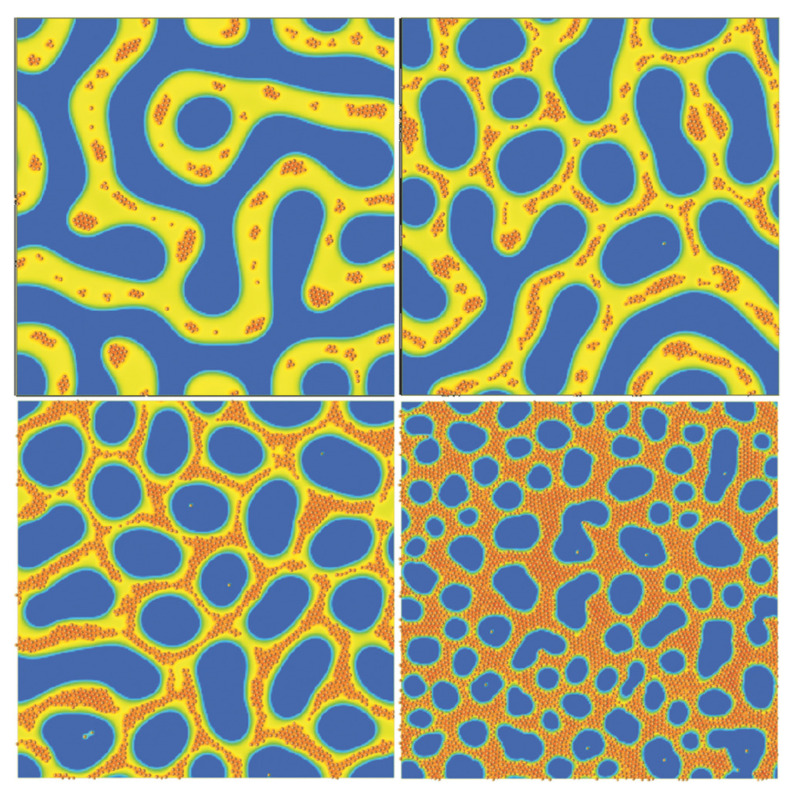
Phase transition for a lamellar BCP morphology and a concentration $\phi_{p}=0.05$, 0.1, 0.2 and 0.5 of NPs, respectively, for top left, top right, bottom left and bottom right. Affinity of the NPs is $\psi_{0}=1.2>\psi_{eq}$, a larger value than the order parameter takes in the absence of colloids. Reprinted with permissions from Ref. [144], Copyright 2017 Wiley.

The vast majority of experimental and computational works on BCP nanocomposites are devoted to NPs which are either miscible within one of the BCP phases or, to a certain degree, anchoring at the interface between blocks. Contrary to that, Shenhar et al. [43] studied gold NPs incompatible with both blocks, but to a different extend. This resulted in the preferential segregation of NPs into the least incompatible domains and, additionally, the formation of hexagonally close-packed NP structures as the NPs are expelled from the matrix. Within the TDGL/BD model, this can be reproduced by introducing affinities which are larger than the equilibrium values of the BCP order parameter $\psi_{0}>\psi_{eq}$ [144]; by doing so, each NP introduces an energetic penalty which is minimised by NP aggregation into hexagonal configuration, as shown in Figure 11. Furthermore, the dynamics of the segregation of NPs into the centre of BCP domains could be studied, finding a slower time scale for the NP segregation than the comparatively faster BCP phase separation. These simulation results are in good agreement with experimental images involving gold NPs [43].

### 3.2. Three-Dimensional Systems

**Figure 12:**
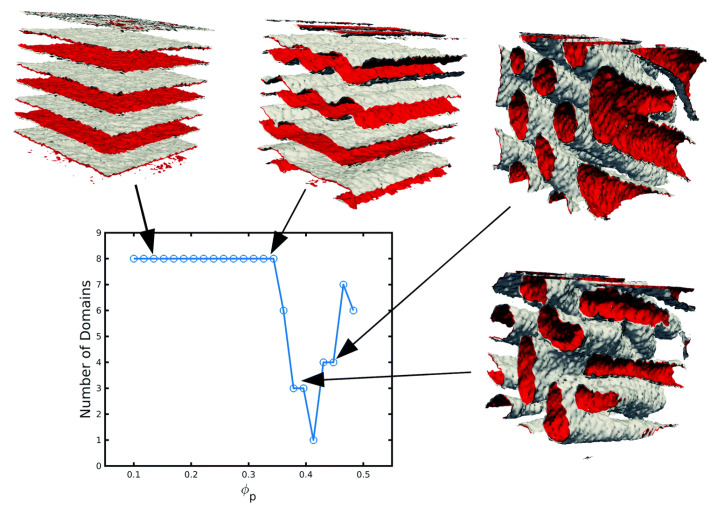
BCP transition from lamellar-forming to hexagonally ordered cylinders, tracked by the number of domains of a symmetric ($f_{0}=1/2$) BCP as a function of the concentration of colloids $\phi_{p}$. Snapshots of the final state of some representative simulations are shown, where the colloids are not shown for clarity. Reprinted with permission from Ref. [182] 2019 Royal Society of Chemistry.

The computational speed of the TDGL/BD approach allows to reach large system sizes in 3D simulations. Furthermore, the richness of the BCP phase diagram is considerably enhanced in 3D, with additional morphologies such as BCC spheres, hexagonally ordered cylinders and a gyroid phase. The 2D transition shown in Figure 10 (lamellar to circles) can be reproduced in 3D: in Figure 12, starting from a lamellar morphology—top left snapshot—due to the symmetry in the BCP chain, the addition of an increasing concentration of particles (not shown for clarity) leads to the BCP transition towards the hexagonally ordered cylindrical phase—bottom right snapshot. The transition in Figure 12 can be tracked via the number of BCP domains in the system: at a low NP concentration the number of BCP domains remains high, indicating the number of lamellar periods. At the onset of phase transition, $\phi_{p}∼0.35$, the lamellar interface fluctuates in order to better accommodate the considerable concentration of NPs. Following that, the lamellar mesophase is broken into a highly connected structure that leads to a reduction in the number of BCP domains which, at concentration $\phi_{p}∼0.5$, completes the transition into a cylindrical morphology organised in a hexagonal lattice. The assembly of selective NPs within curved lamellar domains has been studied experimentally, finding onionlike structures [45,181].

On top of the effect of NPs on the BCP mesophase, NPs can coassemble within the BCP phase to form ordered structures. In the case of simple isotropic NPs compatible with one of the BCP phases, the BCP can act as a soft confinement upon the NPs. As a result, depending on the relative length scales of the BCP spacing and the NP diameter, NPs can form close-packed structures. For instance, in the case of lamellar BCP phases, the NPs assemble into layered structures, as shown Figure 6 in reference [182], where the ordering of NPs is quantified with the hexatic bond-order parameter. Contrary to that, in cylinder-forming BCP, NPs can be segregated within the cylinders, where again the NPs are softly confined and thus assemble into radial onionlike layers, where similarly the number of layers is given by the relative lengths of NP diameter and BCP spacing. These snapshots can be seen in Figure 7 in reference [182].

**Figure 13:**
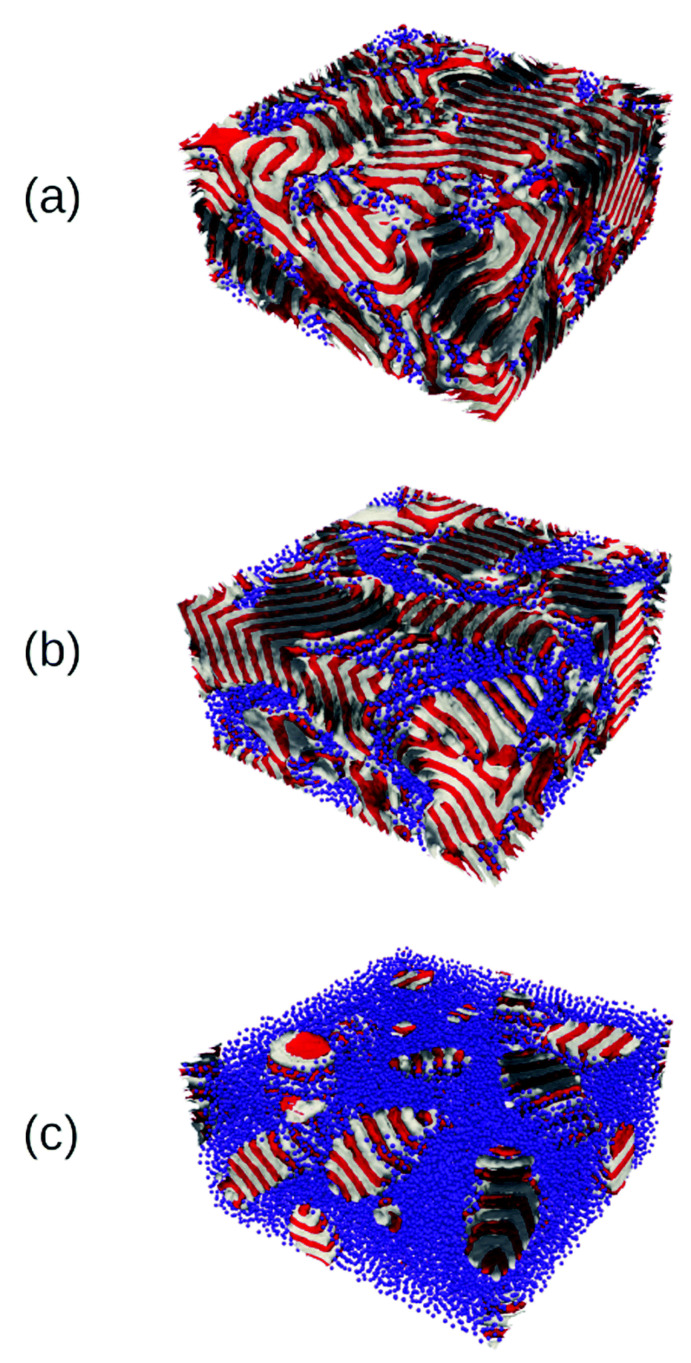
Phase transition of a symmetric ($f_{0}=0.5$) BCP induced by the presence of a concentration $\phi_{p}$ of neutral NPs. The concentrations of NPs are $\phi_{p}=0.1$, 0.24 and 0.45 for (**a**–**c**), respectively. Reprinted with permission from Ref. [182] Copyright 2019 from the Royal Society of Chemistry.

Neutral (interface-compatible) NPs, as shown in Figure 8, were simulated in 3D when dispersed in a lamellar-forming BCP. At a low concentration $\phi_{p}=0.1$ NPs were found to simply segregate towards the interface between A and B domains, as shown in Figure 13a. The NP length compared with the BCP period is considerable with $R/H_{0}=0.3$, which translates into a distortion in the BCP surrounding induced by the presence of each neutral NP, amplified by the weak segregation regime of the BCP. This distortion promotes the aggregation of NPs via an effective attractive potential driven by the minimisation of the distortion, i.e., driven by $F_{cpl}$. This effective interaction is similar to the one observed for incompatible NPs promoting the aggregation into hexagonal close-packed configurations [144] (see Figure 11). As the concentration of NPs grows to $\phi_{p}=0.24$ (Figure 13b) neutral colloids are able to form an almost continuous network of NPs, that is, macrophase separation is occurring. Finally, at moderate concentrations, the phase separation between BCP rich and NP rich is clearly observable, as shown in Figure 13c. Here, a single continuous NP-rich domain is formed, in which isolated BCP-rich dropletlike domains are formed. Interestingly, the shape of such BCP-rich domains is controllable by the NP–BCP interaction, which determines the contact angle between the lamellar domains and the NP–BCP interface. Due to the perpendicular alignment of lamellar domains with the NP interface, the BCP domains are elongated in the direction perpendicular to the lamellar planes. These structures are easily mapped into ternary mixtures of BCP/homopolymer where the interaction between the different components dictates the phase separation and contact angles [183,184]. We note that the slow kinetics of macrophase separation, such as the one shown in Figure 13c, requires the use of efficient computational tools and, for relatively large systems, parallel implementations.

### 3.3. Shape-Anisotropic NPs

**Figure 14:**
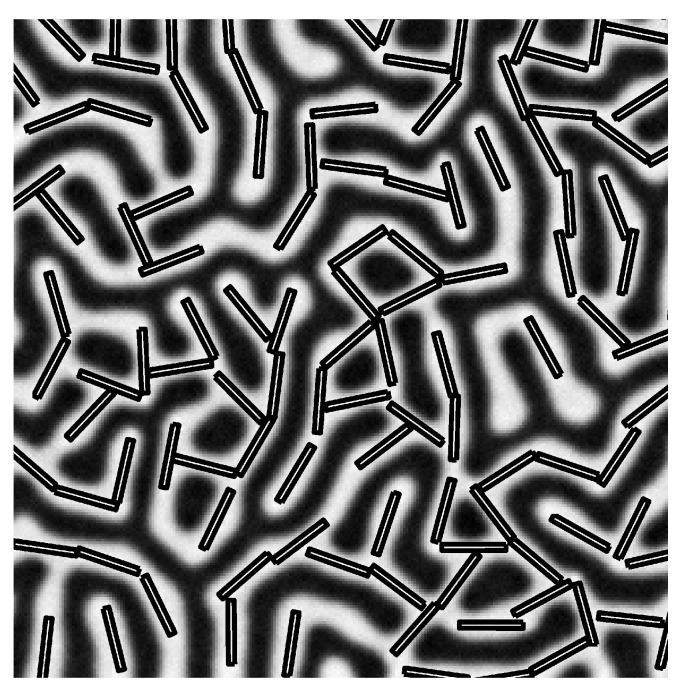
Alignment of rectangular NPs in a symmetric block copolymer. NP size is $a/L_{0}=1.5$ and $b/L_{0}=0.15$ for the major and minor semiaxis, for a lamellar spacing $L_{0}$. Reprinted with permission from Ref. [160] Copyright 2019 American Chemical Society.

In addition to controlling the NP placement within the BCP matrix, anisotropic NPs may also undergo orientational alignment with respect to the BCP structure. Again, given the BCP intrinsic ordering, this potentially allows to obtain a precise control over both the translational and rotational degrees of freedom of complex-shaped NPs. Perhaps the most common realisation of anisotric NPs within BCP matrices are elongated NRs, which may be typically metallic (namely Au NPs) or semiconductive. NRs’ orientation can be experimentally controlled by the BCP lamellar morphology in thin films or cylindrical domains in bulk [106,107,108,109,110]. In both cases, the soft confinement introduced by the BCP templates the alignment of the NRs. By introducing anisotropic parameters in the shape function $\psi_{c}$ in Equations (12) and (18), the NP shape could be explored in terms of the aspect ratio *e* and the super-ellipse exponent *n* [160]. Rectangular-shaped NPs with exponent n=2 and small aspect ratio e=0.1 can be segregated within a lamellar-forming BCP. In Figure 14, on top of the segregation of NPs within one of the BCP components, due to the affinity $\psi_{0}=-1$ towards the white phase, the rectangular NPs are found to align along the lamellar domains to the relatively long length of the larger dimension of the NPs. Additionally, an end-to-end organisation of NPs can be observed, which is in accordance with experimental works [185].

Contrary to relatively long NRs (compared with the BCP periodicity), moderately sized semiconductive NRs have been found to organise side by side when segregated within lamellar-forming BCP melts in ultra-thin films [113,114]. This is due to both the particle–particle attractive interaction, which is maximised in such configuration, as well as the NP interaction with the incompatible phase, which is minimised by NPs arranging perpendicularly to the BCP domains.

**Figure 15:**
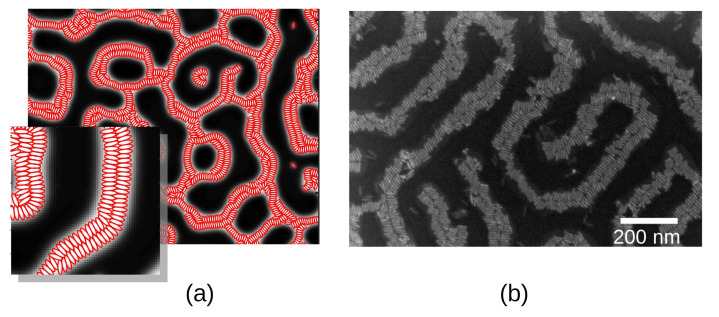
Moderate filling fraction of anisotropic NPs in BCP mixture. Comparison between (**a**) simulations with inset and (**b**) SEM image showing 33 nm-long CdSe NRs coassembled with PS-*b*-PMMA with $H_{0}=132$ nm periodicity (PS domain size is $L_{0}=75$ nm). The experimental NR diameter is 4.6 nm with a filling fraction of 0.26. Reprinted with permission from Ref. [115]. Copyright 2020 American Chemical Society.

In order to capture the attractive component of the potential, leading to the energetic gain upon side-by-side arrangement, a Gay–Berne potential was used, as described in Equation (19). Controlling the energetic depth of the potential well, it was possible to achieve a simulated configuration which is largely equivalent to experiments, as shown in Figure 15a,b, respectively, for simulated ellipsoids and scanning electron microscope (SEM) image of CdSe NRs. The 2D ellipsoid NPs organise in a side-by-side configuration forming two layers. By means of mesoscopic simulations, further insight can be obtained on the time evolution [115]: NPs with a strong NP–NP interaction are highly affected by the initial condition of the NPs, e.g., whether NPs are initially ordered.

**Figure 16:**
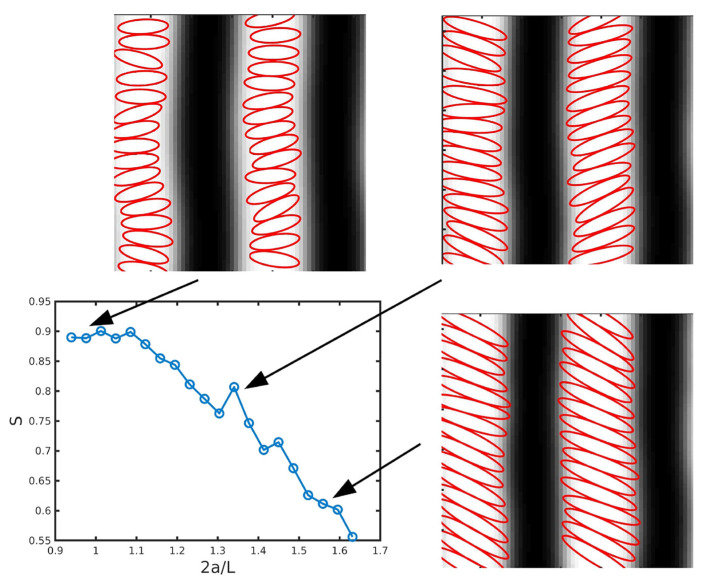
Decrease in orientational ordering of ellipsoids when the size of the major axis 2a is larger than the available normal spacing $L\approx H_{0}/2$ with $H_{0}$ the lamella periodicity. Reprinted with permission from Ref. [115]. Copyright 2020 American Chemical Society.

The alignment of the NR with respect to the lamellar domain strongly depends on the ratio of the NR length with the BCP spacing: relatively long NPs have been shown to align along the BCP domains, as in Figure 14. On the other hand, relatively short NPs may organise perpendicularly to the domain axis, as in Figure 15. For intermediate lengths, NPs were found to undergo a rotation with respect to the domain axis linking these two regimes [115], as shown in Figure 16,which connects these previously studied regimes.

**Figure 17:**
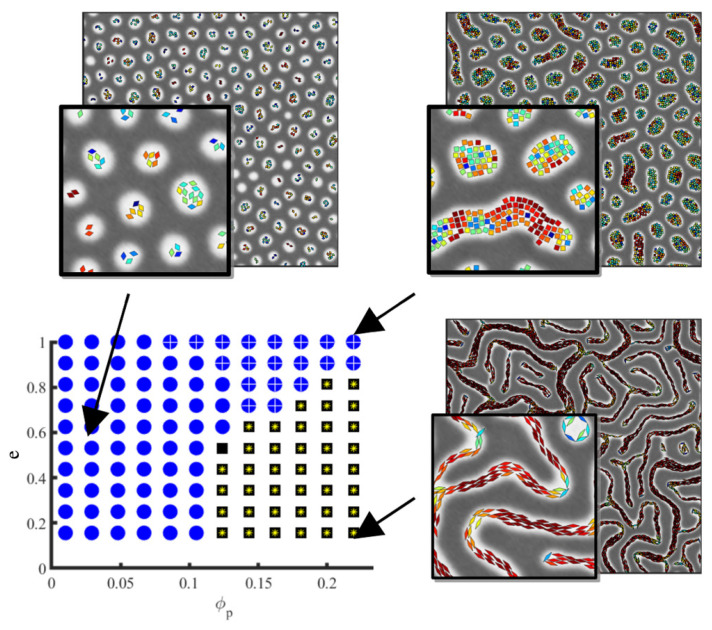
BCP phase behavior depending on the NP anisotropy from squares (*e* = 1) to rhomboids (*e* = 0.2), and a volume fraction $\phi_{p}$ of particles. The BCP phase is marked as blue squares for circular morphology and black squares for lamellar phase. The colloid–colloid alignment is marked by a yellow asterisk for simulations with high twofold nematic order $S_{cc}$ and white crosses for low twofold nematic order but high fourfold tetratic order $S_{cc}^{4}$. NPs are colored according to their local nematic ordering. Reprinted with permission from Ref. [170] Copyright 2022 Wiley.

In the absence of an attractive component in the NP–NP potential, anisotropic NPs do not self-assemble into a side-by-side organisation, even when the NP length is smaller than the lamellar spacing [170]. On the contrary, they are found to align along the direction of the domain axis. This alignment is enhanced in the lamellar morphology and reduced in other phases. Conversely, the presence of anisotropic NPs can additionally modify the BCP morphology, as shown in the phase diagram in Figure 17 from reference [170]. As the rhomboidal-shaped NPs acquire a local nematic ordering, the BCP transitions from a circular phase to a lamellar-like morphology, but only for relatively anisotropic NPs with a small aspect ratio *e*. This indicates a BCP phase transition determined by the NP anisotropy, which has been previously reported in experiments in thin films where the BCP morphology transitions from cylindrical to lamellar in the presence of NRs, but not in the presence of isotropic NPs as shown in Figure 1. These simulation and experimental results suggest the coassembly of anisotropic NPs into BCP matrices, where BCP do not act as mere templates but can be modified by the NP concentration and shape.

**Figure 18:**
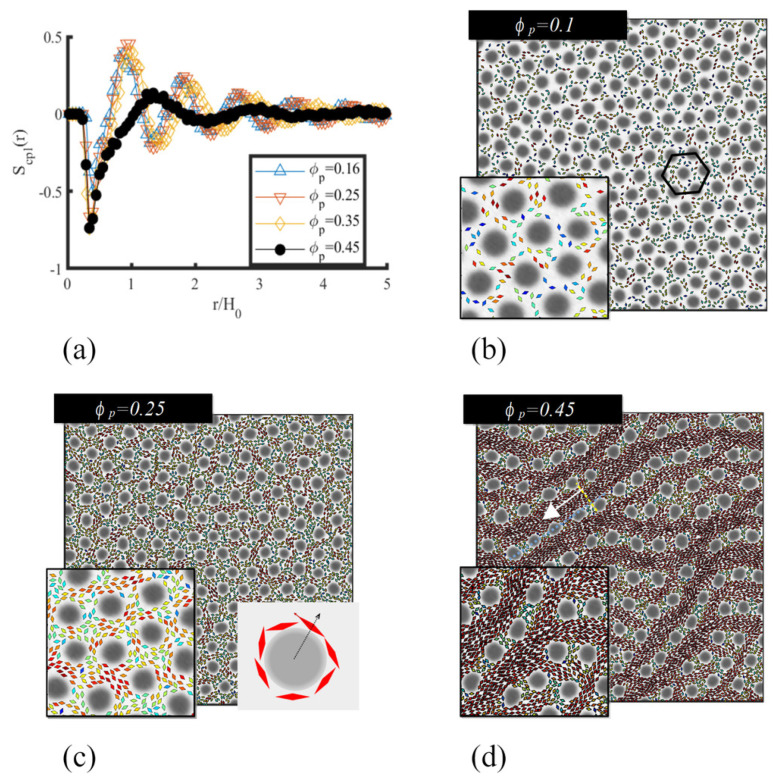
Nematic ordering of rhomboidal NPs (e=1/2) within the majority phase of circle-forming BCP ($f_{0}=0.6$). (**a**) shows the coupling nematic order parameter $S_{cpl}\left(r\right)$ in function of the distance to the center of BCP domains. (**b**–**d**) show the snapshots as the concentration grows, respectively, for $\phi_{p}=0.1$, 0.25 and 0.45. Reprinted with permission from Ref. [170] Copyright 2022 Wiley.

At a low concentration, anisotropic NPs compatible with the majority phase in a circle-forming BCP display no defined order. Instead, they are segregated within the continuous phase, as shown in Figure 18b at a volume fraction $\phi_{p}=0.1$. As the concentration increases, NPs are pushed into the domain walls as a result of the steric interaction between particles. This leads to the tangential organisation of NPs along the BCP interface, as shown visually in Figure 18c (see schematic view in inset). This can be quantified calculating the coupling nematic order parameter $S_{cpl}\left(r\right)$ of NPs with respect to the vector connecting the particle’s centre of mass and the centre of each BCP domain. This can be determined as $S_{cpl}\left(r\right)=\langle S_{cpl}^{i,\nu }\rangle$, as the average over both particles *i* and all BCP domains ν of $S_{cpl}^{i,\nu }=2{\left({\hat{u}}_{i}\cdot {\hat{r}}_{i\nu }\right)}^{2}-1$ where the unit vector ${\hat{r}}_{i\nu }=\left(r_{i}-R_{\nu }\right)/\left|\right|r_{i}-R_{\nu }\left|\right|$ is the relative vector between the NP and the BCP domain center of mass. In terms of the distance *r* to the centre of each BCP domain, we can observe a curve profile that maintains the BCP periodicity, suggesting that the presence of NPs does not distort the mesophase. The curves display a first negative peak corresponding to particles with a tangential alignment with the BCP interface, followed by a positive peak corresponding to the first shell of neatest neighbors to a given BCP domain. Several NP concentrations are seen to collapse into a single curve, with the orientation of NPs clearly templated by the BCP periodicity.

For higher concentrations, the excluded volume interaction between particles dominates, giving rise to local nematic order, similar to the one shown in Figure 17 bottom right. In this regime, the NP tendency towards global nematic order competes with the BCP mesophase, which forms a circular domains in a hexagonal latice. The result of this competition, shown in Figure 18d, is the distortion of the hexagonal lattice into a rectangular configuration controlled by the direction of the local nematic order. Long regions of high nematic order can be seen as red stripes along the simulation box.

**Figure 19:**
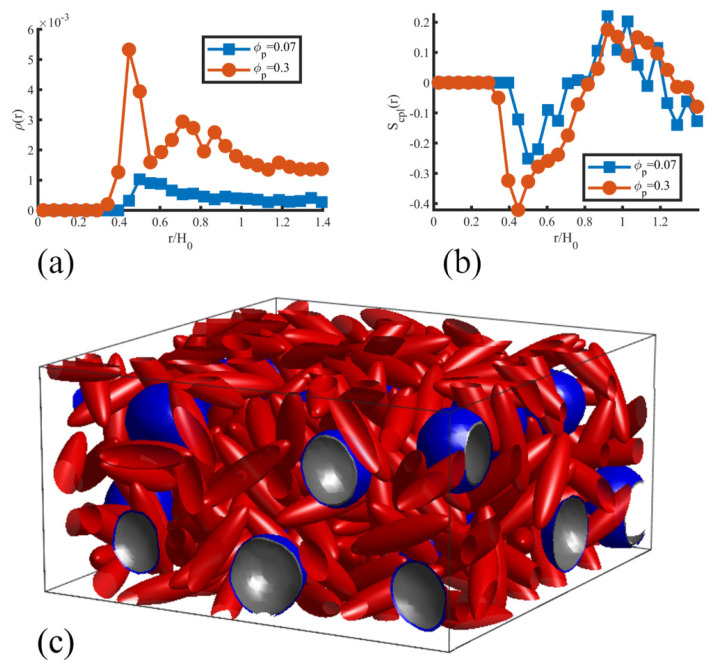
Orientational and translational assembly of 3D ellipsoidal NPs with anisotropy e=1/4 in a BCC sphere forming BCP with $f_{0}=0.7$. NPs are miscible in the majority (continuous) phase. (**a**,**b**, respectively, show the curves for the particle density $\phi_{p}$ and coupling nematic order parameter $S_{cpl}\left(r\right)$ with respect to the radial vector to the center of BCP domains in function of the distance *r* to the center of BCP domains). In (**c**) a snapshot of a dense simulation $\phi_{p}=0.3$ is shown, with NPs shown in red and the isosurfaces of the BCP interface shown in blue and grey. Reprinted with permission from Advanced Theory and Simulations, 2022, 5(1), 2100433 [170] Copyright 2022 Wiley.

A similar behavior was found in 3D by exploiting the ability to simulate 3D nonspherical particles following Equation (18). Ellipsoids interacting with a modified Yukawa interaction, as in Equation (20), could be found to organise nematically with respect to the BCP interface within a first shell (density peak in Figure 19a) placed at the walls of the BCC sphere BCP domains. As anisotropic NP concentration grows, the repulsive interaction between particles leads to the alignment of NPs when exposed to the domain walls. Again, this translates into a nematic order $S_{cpl}\left(r\right)$ calculated with respect to the centre of BCC spherical domains, reminiscent of paranematic ordering under confinement [186]. The likeness in the behaviour of anisotropic NPs in Figure 18 and Figure 19 suggest the generality of the coassembly process, regardless of the specific NP shape (rhomboids and ellipsoids) or the dimensionality of the system (2D and 3D).

In addition to the phase transition shown in Figure 17, recent 3D simulations show that the presence of large anisotropic NPs can influence the morphology of the BCP by promoting a cylindrical phase against a BCC sphere phase, which is considerably more isotropic. This has been shown to occur for extremely low concentrations, well below the isotropic–nematic phase transition, and when NPs are compatible with the majority phase [187]. These results, along with Figure 17, suggest that the determination of the phase of a BCP/anisotropic NP system need to take into account both the ability of NPs to organise nematically (short NPs, high concentrations) and to induce nematic order in the BCP (long NPs and low concentrations). This is, in fact, a rather general result, and NPs have been shown to promote nematic order in liquid crystals [188].

### 3.4. Chemically Inhomogeneous NPs

**Figure 20:**
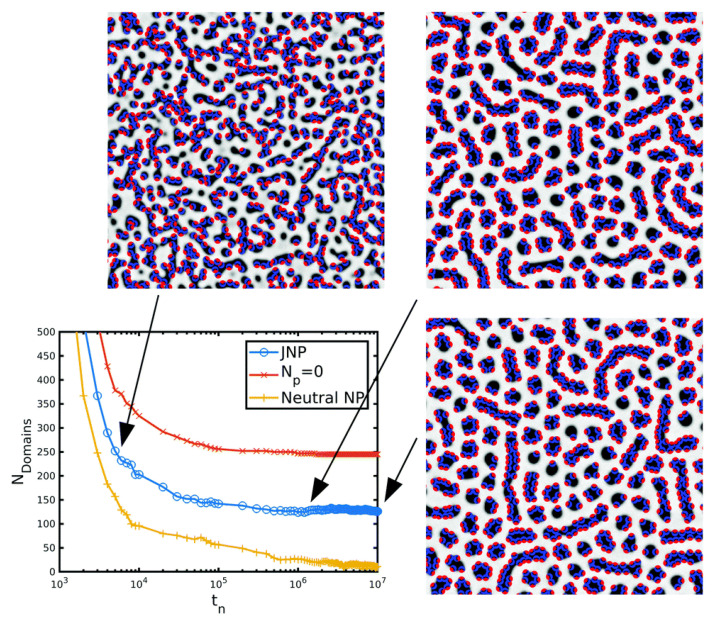
Time evolution of the number of BCP domains in the presence of JNPs, no particles and homogeneously coated NPs, along with several images of the simulation results. The pure BCP is cylinder-forming ($f_{0}=0.35$) while the presence of the JNP favours domain merging. The curves for pure BCP ($N_{p}=0$) and homogeneous neutral NPs are also shown for comparison. Reprinted with permission from Ref. [171] Copyright 2019 Royal Society of Chemistry.

Chemically inhomogenous NPs such as JNPs were simulated following the split-up approach described in Section 2.7. In the simplest possible case, amphiphillic-like JNPs, in which each side of the particle is compatible with one of the blocks of the BCP, behaved as surfactants, segregating towards the interface between BCP domains [171]. Furthermore, if each side of the JNP is completely compatible with each one of the BCP phases, particles acquire a defined perpendicular orientation with respect to the interface, exposing each side to the preferred phase of the BCP. This can be seen in the snapshots in Figure 20, in function of time. The segregation and orientation of JNPs within BCP interfaces has been experimentally observed [142] and reproduced using molecular dynamics [189].

In Figure 20, the formation of the circle-forming BCP mesophase is characterised by the number of BCP domains in the system, in the presence of JNPs, no NPs and neutral homogeneous NPs (ie, as described in Figure 8). It is clear that the presence of NPs, and their chemical composition, modifies the mesophase formation: the addition of NPs of any kind promotes the formation of more connected BCP domains, which translates into a reduced number of domains in the system. This is a consequence of the ability of interface-segregated NPs to form bridges connecting two or more domains. Interestingly, this effect is enhanced in the case of homogeneous neutral NPs: JNPs are more strongly energetically trapped at the interface than their homogeneous counterparts. Furthermore, JNPs have a defined orientation within the interface, which limits the ability to form bridges between BCP domains.

The BCP mesophase was found to change significantly for interface-anchoring homogeneous NPs, while the lamellar structure was preserved in the presence of chemically homogeneous JNPs in Figure 5 in ref. [171]. The increased interface activity of the JNPs when compared with their homogeneous counterparts indicates that JNPs can be suitable candidates to be segregated into BCP interfaces while minimising the effect on the BCP mesophase itself.

Despite JNPs being less disruptive on the BCP mesophase, a high concentration of JNPs may break the BCP domains and increase the overall number of domains, as shown in Figure 20 in the text, and Figure 7 in reference [171]. These are examples of perfectly antisymmetric JNPs in which each side of the JNP is completely compatible with one of the BCP phases.

**Figure 21:**
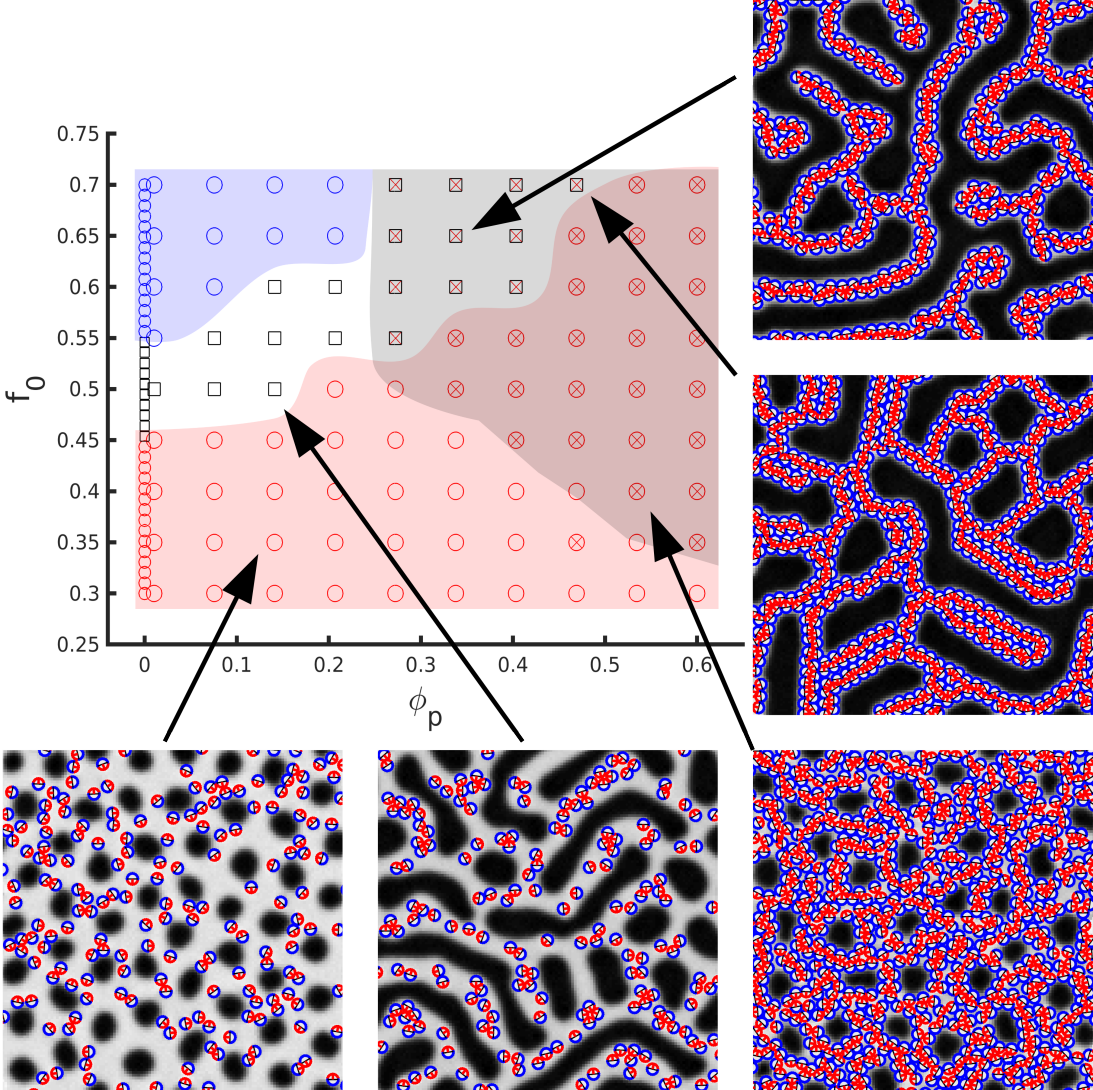
Phase behaviour of the BCP in the presence of a concentration $\phi_{p}$ of JNP with ${\bar{\psi }}_{0}$ = 1.0 and $\Delta \psi_{0}=1.0$. Symbols are as follows: circles stand for cylindrical phase with colour determining the majority (red circles indicate white monomer as the majority and blue circles indicate the opposite); lamellar phase is denoted by black squares; a further cross indicates a high degree of particle-to-particle orientational order. Reprinted with permission from Ref. [171] Copyright 2019 Royal Society of Chemistry.

Contrary to that, in the case of off-center JNPs (${\bar{\psi }}_{0}=1$ and $\Delta \psi_{0}=1.0$, in the parameters expressed in Equation (22)), at low concentration particles are segregated within one of the BCP phases, as shown in Figure 21 bottom-left snapshot. Here, one side (red) of the JNP is compatible with the white phase, while the other one (blue) is compatible with the interface. This leads to the segregation of JNPs within the white phase. Even though there is a negligible energetic gain by JNPs arranging in an organised manner (see Figure 4 in reference [171]), this structure is largely destroyed by thermal motion. On the other hand, at a high concentration and high confinement ($f_{0}>0.5$), two processes occur: the BCP undergoes a circle-to-lamellar transition and JNPs are forced into the interface due to high local packing fraction. In order to maximise the exposure of compatible sides of the JNP into the BCP, the JNPs acquire a lamellar-like structure formed by an even number of layers (2 and 4 layers in top-right and middle-right snapshots, respectively). The JNPs expose the blue side to the interface while the red side is wetted by the white phase.

## 4. Concluding Remarks

The TDGL scheme has been successfully applied to a wide variety of equilibrium and out-of-equilibrium BCP systems. This review has covered recent developments in TDGL models coupled with BD that result in an efficient hybrid in-grid/out-of-grid scheme. The considerable computational speed of the scheme, as well as the relatively easy extension into complex systems, makes TDGL/BD a suitable model for mesoscopic simulations of BCP nanocomposites. This is largely due to the simplicity of the model that coarse grains the microscopic details of the polymer chain and simplifies the BCP–NP coupling to essentially harmonic interaction.

The computational efficiency of the TDGL/BD, as well as its ability to take advantage of HPC facilities through paralelisation, has allowed to scale up to considerably large system sizes for representative times. Being able to simulate such large systems can be crucial to reproduce experimental setups which may be trapped in metaestable states, frequent in soft matter systems. Additionally, several of the mechanisms described in this work posses both faster and slower time scales, the latter being typically associated with global ordering (for example, BCP morphology acquiring system-wide order, macrophase separation, or emergence of nematic order). To address the slower dynamics which often appear in soft matter systems, the TDGL/BD model is a suitable tool, which may be used in tandem with more microscopically accurate models such as molecular dynamics or Monte Carlo.

Despite its simplicity, which neglects many of the microscopic degrees of freedom present in the system, TDGL/BD has reproduced many experimental setups and behaviours. Representative examples include: NP induced phase transition [57], hexagonal close packed ordering of NPs [144], alignment of anisotropic NPs [115,160] and anisotropy-induced transition [170] in the BCP and alignment of JNPs [171].

This review has devoted attention to the recent extensions of hybrid TDGL/BD models with single-integral coupling for complex NPs. Such models address the increased use of nonspherical NPs for experimental setups and has been shown to correctly reproduce the formation of hierarchical structures (see Figure 15). Additionally, comparisons have been drawn with alternative approaches to incorporate NP anisotropy in the coupling free energy. Due to the simplicity of the model, the specific polymer–particle interaction is not expected to play a fundamental role, as long as the chemical selectivity is introduced and the NP shape is incorporated. On the contrary, the particle–particle interaction may play a crucial role in determining the assembly of anisotropic NPs, which in turn can affect the coassembly of the mixture. A clear example of this is the contrast between the high concentration of anisotropic NPs with and without attractive interaction between NPs, shown for example in Figure 15 and Figure 17, respectively. This is not a purely theoretical contrast, as semiconductive NRs may exhibit permanent dipoles resulting in attractive interactions when compared with metallic NRs [115].

The TDGL/BD scheme could be easily extended to simulate more complex systems, such as a thin films with isotropic or anisotropic NPs, where the particle–surface interaction may play a role in the alignment of NPs [190]. Recent experimental setups involve anisotropic NPs with shapes other than NRs [116]. The richness of self-assembly behaviour for generic nonspherical NPs [100,191] could motivate the use of TDGL/BD as a precursor to explore the complex phase behaviour of NPs with various shapes.

## Data Availability

This study did not report any new data.
